# Improvement in Hypertension Control Among Adults Seen in Federally Qualified Health Center Clinics in the Stroke Belt: Implementing a Program with a Dashboard and Process Metrics

**DOI:** 10.1089/heq.2022.0109

**Published:** 2023-02-08

**Authors:** Edward M. Behling, Tammy Garris, Vicky Blankenship, Shaun Wagner, David Ramsey, Rob Davis, Susan E. Sutherland, Brent Egan, Gregory Wozniak, Michael Rakotz, Karen Kmetik

**Affiliations:** ^1^ HopeHealth, Florence, South Carolina, USA.; ^2^ American Medical Association, Greenville, South Carolina, USA.; ^3^ American Medical Association, Chicago, Illinois, USA.

**Keywords:** hypertension, health equity, quality improvement, dashboard

## Abstract

**Objective:**

Attain 75% hypertension (HTN) control and improve racial equity in control with the American Medical Association Measure accurately, Act rapidly, Partner with patients blood pressure (AMA MAP BP™) quality improvement program, including a monthly dashboard and practice facilitation.

**Methods:**

Eight federally qualified health center clinics from the HopeHealth network in South Carolina participated. Clinic staff received monthly practice facilitation guided by a dashboard with process metrics (measure [repeat BP when initial systolic ≥140 or diastolic ≥90 mmHg; Act [number antihypertensive medication classes prescribed at standard dose or greater to adults with uncontrolled BP]; Partner [follow-up within 30 days of uncontrolled BP; systolic BP fall after medication added]) and outcome metric (BP <140/<90). Electronic health record data were obtained on adults ≥18 years at baseline and monthly during MAP BP. Patients with diagnosed HTN, ≥1 encounter at baseline, and ≥2 encounters during 6 months of MAP BP were included in this evaluation.

**Results:**

Among 45,498 adults with encounters during the 1-year baseline, 20,963 (46.1%) had diagnosed HTN; 12,370 (59%) met the inclusion criteria (67% black, 29% white; mean (standard deviation) age 59.5 (12.8) years; 16.3% uninsured. HTN control improved (63.6% vs. 75.1%, *p*<0.0001), reflecting positive changes in Measure, Act, and Partner metrics (all *p*<0.001), although control remained lower in non-Hispanic black than in non-Hispanic white adults (73.8% vs. 78.4%, *p*<0.001).

**Conclusions:**

With MAP BP, the HTN control goal was attained among adults eligible for analysis. Ongoing efforts aim to improve program access and racial equity in control.

## Introduction

The excess stroke mortality rate in the southeast United States led to its designation as the nation's Stroke Belt.^1,2^ For all but one decade between 1930 and 1990,^3^ South Carolina had the highest per capita stroke mortality rate in the southeast, that is, the “buckle” of the Stroke Belt.^4^ Within South Carolina, the Pee Dee Region, located in the northeast section of the state, has a very high rate of stroke-related deaths.^5^ HopeHealth, designated as a federally qualified health center (FQHC) in 2007, has 15 clinical sites in the Pee Dee Region of South Carolina serving a primarily rural area with multiple sociodemographic risk factors for adverse cardiovascular outcomes.^6^

HopeHealth established goals to attain a hypertension (HTN) control rate of 75% and improve racial equity in control before discussions with the American Medical Association (AMA). Previous experience documented that the AMA MAP BP™ program (Measure accurately, Act rapidly, Partner with patients) could enable a rapid and sustained improvement in HTN control within clinical sites serving patients with sociodemographic risk for HTN-related morbidity and mortality rate.^7–9^

Given the burden of HTN in our patients, HopeHealth partnered with the AMA on their MAP BP quality improvement program.^7^ The eight clinical sites at HopeHealth, which provided primary care to ∼45,000 adults with encounters in the past year, participated in the MAP BP program described in this report. The other seven HopeHealth sites, which did not participate in MAP BP, predominantly provide medical services to children or other adult nonprimary care medical services.

The MAP BP program includes monthly dashboards and practice facilitation to promote efficient implementation and maintenance of key process changes that raise HTN control.^9^ The monthly dashboards with process metrics and HTN control metric at the clinic level were provided to the eight HopeHealth sites individually, and at the patient level for providers at each clinical site. This current report presents changes in HTN control, blood pressure (BP, mmHg), and process metrics during the 6-month MAP BP quality improvement program.

## Methods

### Ethical and regulatory considerations

MAP BP was designated a quality improvement program and exempted from oversight by the AMA's Institutional Review Board of Record. The HopeHealth–AMA collaboration was conducted under a Business Associate Agreement, which allows exchange of personal health information required for quality improvement, and Data Use Agreement, which permits use of a limited data set for analysis and reporting.

### Inclusion criteria

Eligible patients had a diagnosis of essential HTN defined by ICD-10-CM I10, before program implementation and at least one recorded BP during the 1-year baseline period, defined as February 1, 2020, to January 31, 2021. Eligible individuals also had at least two encounters with recorded BP during the 6-month MAP BP intervention from March 1, 2021, to August 31, 2021. Adults not meeting these criteria were excluded from the main analysis.

### Implementation of the MAP BP quality improvement program

MAP BP began with virtual clinical site assessments at three HopeHealth locations to understand clinical workflows and processes related to BP measurement and HTN management.^7^ Separate 1-h training periods for physicians and other providers and care team members provided a program overview, the importance of M, A, and P processes in improving BP control, and an overview of the metrics and dashboard. Clinical care team members received an additional 1-h workshop and an hour-long orientation on the MAP BP dashboard. Approximately 13 h of virtual practice facilitation were then provided on M, A, and P by the AMA team members concurrently to participating clinical sites through tele-video conferences.

The goals of practice facilitation were to provide clinical education, program implementation support, review monthly data, and assist with logistical problem-solving required to efficiently incorporate MAP BP into daily operations at each site. Physicians, advanced practice nurses, clinical pharmacists, and physician assistants received a 45-min training on managing apparent treatment-resistant HTN.^10,11^

### Data acquisition

Two years of historical data were extracted on adults ≥18 years old from the enterprise data warehouse, which contained data from the Epic electronic health record system used by all HopeHealth clinical sites, before implementing MAP BP. Data were obtained monthly during implementation to enable monthly updating of dashboards and reports.

### Definitions and measurements

*Hypertension* was defined by a diagnosis in the medical record of ICD-10 codes I10–I16 during the 2 years before program implementation. *Hypertension control* was defined by team-measured or automated office (AO)BP <140/<90 based on the most recent value during the 12-month baseline and at the most recent visit during MAP BP.

### BP measurements

*During the baseline*, BP was measured according to usual practice at each site but did not include AOBP. HopeHealth promoted repeat BP measurement when the initial BP values were ≥140 systolic or ≥90 diastolic during the baseline.

*BP measurements during MAP BP: Initial attended BP:* Clinical care team members were trained to measure BP using proper patient preparation, positioning, and correct measurement technique.^12,13^ The measurement protocol was to obtain a single BP after the patient was seated for 5 min in a semiprivate area with values recorded in the electronic health record. Initial attended BP values ≥140 systolic or ≥90 mmHg diastolic led to recommendation for a confirmatory measurement, mostly with unattended AOBP. AOBP devices were available at all clinical sites.

*Unattended AOBP* was conducted in the patient's examination room or other private locations.^13,14^ Without additional patient rest, a clinic team member applied the upper arm cuff of the Welch Allyn Connex Spot, activated the device, and left the room. Once activated, the device obtained three AOBP measurements at 1-min intervals with the patient alone. When the AOBP measurements were completed, the team member returned and documented the mean of AOBP values in the electronic health record.

Health care insurance was determined by the primary payer source for adults with HTN and grouped as Medicare, Medicaid, private, and uninsured.

Body mass index (kg/m^2^) was calculated from the most recent height and weight during the baseline period and categorized as underweight or normal (<25), overweight (25–29.9), or obese (≥30).

Race and ethnicity were assessed by patient self-report during initial registration and annual updates. Patients have the option of entering race/ethnicity information into a kiosk or paper form at initial registration and annual updates. The patient information is then entered by reception staff into the electronic medical record. Patients were categorized as Hispanic ethnicity of any race, non-Hispanic black, non-Hispanic white, other, or unknown.

Comorbidities were defined by the International Classification of Diseases (ICD-10 CM codes) in the electronic health record data for diabetes mellitus (E10, E11, E13); chronic kidney disease (N18.3, N18.4, N18.5, N18l.6, N18.9, R80.9); and cardiovascular disease (G45.1-G46.8, I20.X–I25.X I46.X, I50.1–I50.9, I61.X, I63.X, I65.X, I66.X, I67.2, I67.8, I67.9, I68.8, I69.1, I69.2, I69.3, I69.8, I69.9, I70.X, I71.X, I72.0, I72.6, I73.89, I73.9, I74.X, I75.X, I79.0, K55.1, Z86.73, Z95.1, Z95.5, Z95.820, Z98.61, Z98.62).

### Key process variables

*Measure accurately* was assessed in adults with HTN as the proportion of visits with a confirmatory measurement relative to the number of visits with an initial attended BP ≥140 systolic or ≥90 diastolic.

*Act rapidly* was defined by therapeutic intensity as the number of antihypertensive medication classes prescribed at standard dose (half the recommended maximum dose) or greater to adults with uncontrolled HTN during the last visit of the baseline period and MAP BP program. The Act rapidly metric contrasts with previous reports,^8,9^ and was based on evidence that therapeutic inertia is persistently high^15–18^ and (1) ∼80% of the BP response to most antihypertensive medications occurs at standard or half-maximal dose,^19^ (2) a mean of ≥3 antihypertensive medication classes was required in clinical trials to attain strict control,^20^ and (3) <20% of adults with uncontrolled HTN in community-based clinics were prescribed three different BP medication classes at standard dose or higher.^21^

*Partner with patients* was assessed with two metrics: (1) changes in systolic BP in the 10- to 180-day window, but no later than August 31, 2021, after each therapeutic intensification, and (2) the percent of patients with a follow-up BP obtained within 30 days of an encounter with systolic BP ≥140 or diastolic BP ≥90 documented in the electronic health record.^8,9^ The reduction in systolic BP after intensification was attributed to the time-period in which the follow-up measurement was made. Process metrics were reported primarily at monthly intervals with 12-month rolling averages available.

### Monthly dashboards

**FIG. 1.**
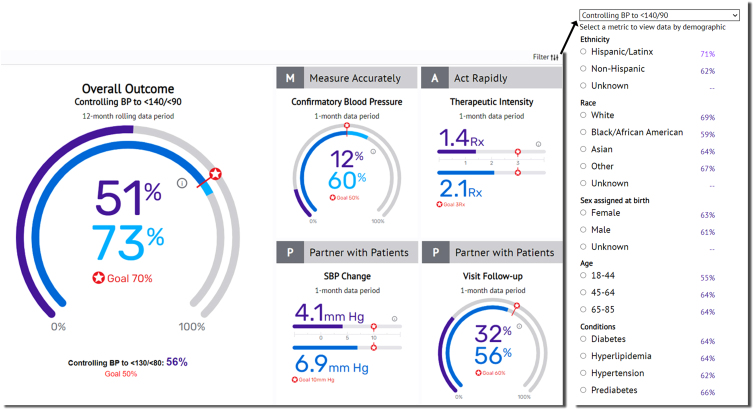
A sample dashboard (fictitious data) provides data for HTN control (outcome metric) and process metrics. The *purple dial* or *bar* depicts data for the individual clinician or clinical site, whereas the *blue dial* or *bar* depicts data for all clinicians (at the site or all sites within the health system), respectively. The *sidebar* (*right*) provides data for demographic subsets or comorbid conditions at the clinician, clinic, or health system level as determined by user authorization. HTN, hypertension.

Physician and provider champions, clinical care team champions, and administrative champions were registered for the dashboard and had access to the information described below. All registered users had access through a secure website to summary data for HTN control and the process metrics (Fig. 1) appropriate for their role. Senior leadership had access to data on HTN control and the four process metrics for the eight clinics collectively and individually.

**FIG. 2.**
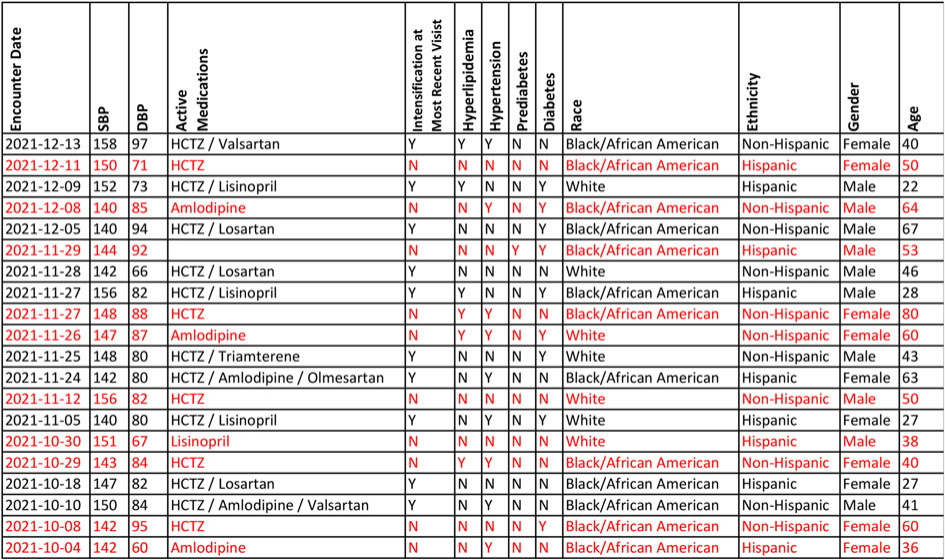
A fictitious patient-level report showing patient name, encounter date, BP values, active antihypertensive medications, demographic details, and comorbid conditions is updated monthly. The list is sortable, for example, uncontrolled HTN or systolic BP (highest to lowest) to facilitate population management for better BP control. BP, blood pressure.

Leadership at each clinical site had data for clinicians at that site collectively and individually, while clinicians had access to data for their patients collectively and individually (Fig. 2). Patient-level reports on BP control and the four process metrics required two-factor authentication and were delivered through secure e-mail to registered users. Patient-level reports listed all patients in the denominator and distinguished by font color those who did not meet the metric objective (Fig. 2). Physicians, advanced practice nurses, physician assistants, and clinical pharmacists could compare their performance with other prescribers at their site or for all participating HopeHealth sites.

### Statistical analysis

Descriptive statistics were used to summarize baseline demographic and clinical characteristics of adults with HTN. Data are reported as relative frequency or mean and 95% confidence intervals. The primary outcome variable was change in HTN control from baseline to the most recent visit during the 6-month MAP BP program among adults in the primary analysis. Changes in MAP BP process variables comprised the secondary outcome. Sensitivity analysis included all patients in the baseline period who had the following: (1) one or more MAP BP visits and (2) irrespective of the number of MAP BP visits, that is, last baseline observation carried forward for those without MAP BP visits.

Pooled *t* and chi-square tests were performed to assess differences in demographic and clinical characteristics between patients included and excluded in the analysis, and for comparisons of patient groups. Satterthwaite's adjustment was used in pooled tests when group variances were not equal. Effect modification of race/ethnicity differences between the baseline and implementation periods was examined with analysis of variance models including the two main factors of time-period and race/ethnicity and their interaction. Paired *t* tests and McNemar tests were used to assess longitudinal outcomes and process measures related to M, A, and P within the non-Hispanic black and non-Hispanic white patient groups. All analyses were performed with two-tailed tests using SAS/STAT software.

## Results

Table 1 compares adults who were eligible and ineligible for the main evaluation. Adults eligible for analysis were older, more likely to be female and non-Hispanic black, and had a higher prevalence of obesity, diabetes, chronic kidney disease and cardiovascular disease than those excluded from the main analysis (all *p*<0.001). Of note, 16.3% of adults with HTN were uninsured.

**Table 1.** Selected Characteristics of Adults with a Diagnosis of Hypertension

Variable group	All patients with HTN	MAP eligible^a^	Ineligible
No.	20,963	12,370	8593
Age (years), mean	58.2 [57.94, 58.46]	59.5 [59.10, 59.87]	56.3 [56.04, 56.65]
<45, %	16.6% [16.12, 17.12]	13.1% [12.53, 13.72]	21.6% [20.77, 22.52]
45–64, %	50.1% [49.42, 50.77]	50.7% [49.85, 51.61]	49.2% [48.13, 50.25]
≥65, %	33.3% [32.64, 33.92]	36.1% [35.30, 36.99]	29.2% [28.20, 30.12]
Female, %	60.9% [60.24, 62.56]	63.8% [62.99, 64.68]	56.7% [55.64, 57.73]
Non-Hispanic black, %	65.4% [64.79, 66.07]	67.4% [66.53,68.18]	62.7% [61.63, 63.68]
Non-Hispanic white, %	30.3% [29.72, 30.97]	29.0% [28.17, 29.76]	32.3% [31.34, 33.32]
Hispanic, %	2.4% [2.22, 2.64]	2.3% [1.99, 2.52]	2.7% [2.35, 3.03]
Other, %	1.1% [0.91, 1.19]	0.8% [0.68, 1.00]	1.4% [1.11, 1.59]
Unknown, %	0.7% [0.63, 0.86]	0.6% [0.45, 0.72]	1.0% [0.77, 1.19]
Body mass index (kg/m^2^), mean	32.9 [32.82, 33.06]	33.2 [33.00, 33.32]	32.6 [32.43, 32.81]
<25, %	17.5% [16.96, 17.99]	16.6% [15.92, 17.23]	18.8% [17.96, 19.61]
25–29.9	24.2% [23.66, 24.82]	23.7% [22.92, 24.42]	25.1% [24.15, 25.98]
≥30, %	58.3% [57.61, 58.95]	59.8% [58.89, 60.62]	56.2% [55.10, 57.20]
Diabetes mellitus, %	37.7% [37.02, 38.33]	43.5% [42.62, 44.37]	29.3% [28.34, 30.27]
Chronic kidney disease, %	17.6% [17.04, 18.07]	21.5% [20.73, 22.18]	11.9% [11.25, 12.63]
Cardiovascular disease, %	12.2.% [11.74, 12.63]	13.7% [13.06, 14.28]	10.0% [9.41, 10.68]

^a^ MAP eligible defined as patients with a previous diagnosis of HTN, at least one clinical encounter during the baseline, and at least two clinical encounters during MAP BP.

Data are reported as percent or mean and 95% confidence intervals.

HTN, hypertension; MAP BP, Measure accurately, Act rapidly, Partner with patients blood pressure.

Changes in key variables between baseline and MAP are shown in Table 2. Systolic and diastolic BP fell as BP control rose from 63.6% at baseline to 75.1% after 6 months of MAP BP (+11.5%, *p*<0.001). The sensitivity analysis (not shown) found that HTN control rose from 63.2% at baseline to 73.7% (+10.5% *p*<0.001) among 16,590 adults with a baseline visit and at least one visit during MAP BP. Among all the 20,370 adults with a baseline visit, irrespective of follow-up, HTN control rose from 61.7% at baseline to 70.2% during MAP BP (+8.5%, *p*<0.001).

**Table 2.** Comparison of Selected Variables During Baseline Versus Measure Accurately, Act Rapidly, Partner with Patients Blood Pressure (*N*=12,370)

Variables	Baseline	MAP 6 Months
Systolic BP (mmHg), mean	134.8 [134.50, 135.13]	129.9 [129.58, 130.18]
Diastolic BP (mmHg), mean	80.2 [80.02, 80.38]	77.3 [77.11, 77.45]
BP <140/<90, %	63.6% [62.72, 64.41]	75.1% [74.31, 75.84]
BP <130/<80, %	26.8% [26.08, 27.64]	38.8% [37.90, 39.61]
Repeat BP, %	39.5% [38.50, 40.51]	48.6% [47.47, 49.81]
Repeat BP ΔSBP (mmHg), mean	10.8 [10.52, 11.12]	11.0 [10.63, 11.26]
Prescribed ≥1 BP medication, %	93.7% [93.27, 94.13]	94.6% [94.22, 95.01]
Therapeutic intensity, mean	1.78 [1.76, 1.81]	1.91 [1.88, 1.94]
No. of BP medications, mean	2.34 [2.32, 2.37]	2.44 [2.42, 2.47]
ΔSBP Rx Int (mmHg), mean	13.8 [13.12, 14.52]	16.9 [15.05, 17.69]
30-Day follow-up, %	23.0% [22.09, 23.86]	29.0% [27.93, 30.14]

Data are reported as percent or mean and 95% confidence intervals.

Repeat (AO)BP with initial value ≥140 systolic or ≥90 diastolic. Therapeutic Intensity, number of BP medication classes prescribed at standard dose or higher for uncontrolled BP. ΔSBP per treatment intensification, mean change in systolic BP when antihypertensive medication class added for uncontrolled BP; 30-day follow-up visit after uncontrolled BP.

*M*easure accurately, assessed by the proportion of adults with a confirmatory measurement when initial BP was uncontrolled improved (+9.1%, *p*<0.001). Systolic BP with repeat measurement was similar at ∼11 mmHg below the initial value during baseline and MAP BP.

*A*ct rapidly, defined by therapeutic intensity in adults with uncontrolled HTN, increased. The mean number of BP medication classes prescribed at any dose rose from 2.45 [2.43, 2.48] at baseline to 2.62 [2.58, 2.65], during MAP BP (data not shown).

**FIG. 3.**
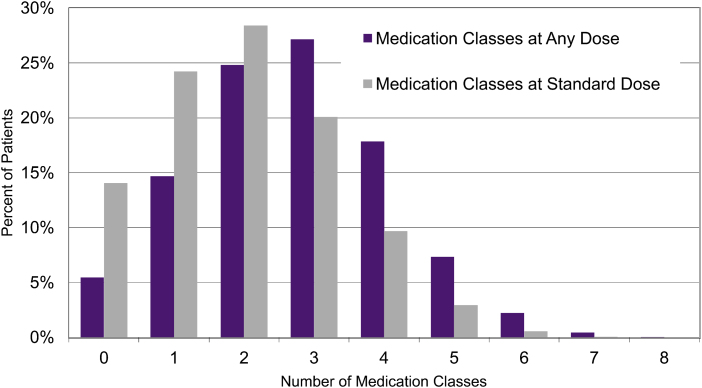
A frequency histogram shows the percentage of adults with uncontrolled HTN who are prescribed a given number of antihypertensive medications at standard dose or higher or any dose. In general, adults with uncontrolled HTN prescribed 0–2 BP medication classes at standard dose are candidates for an additional antihypertensive medication class, whereas the subset prescribed 3 or more medications often merits evaluation for (apparent) treatment resistance before adding medication.

The number of antihypertensive medication classes prescribed at any dose and at standard dose or higher for adults with uncontrolled HTN is shown in Figure 3. The percentages prescribed ≥3 BP medication classes at standard dose versus. any dose or higher where roughly one-third versus 9 of 16.

**FIG. 4.**
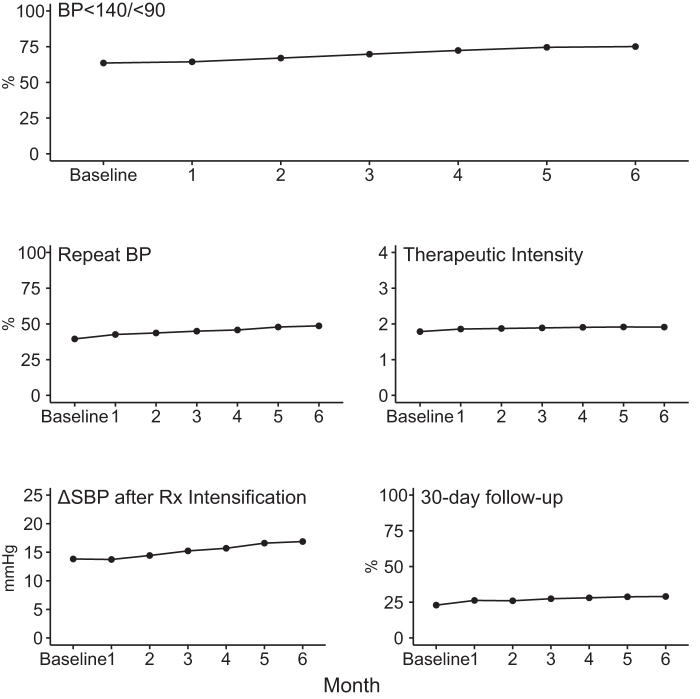
Monthly changes during the 6-month MAP BP intervention in HTN control and the process metrics are shown for the 12,370 adults in the main analysis. Time trends for HTN control and all process metrics were statistically significant at *p*<0.0001.

Partner with patients metrics included the (1) decline in SBP after adding a new antihypertensive medication class, a proxy for adherence, rose from 13.8 to 16.9 mmHg, and (2) percentage of patients having a return visit within 30 days of an uncontrolled BP rose from 23% to 29%. Monthly changes from baseline in outcome and process metrics are shown (Fig. 4).

Significant improvements were seen within the non-Hispanic black and non-Hispanic white patient groups for BP control and process metrics (Table 3). Therapeutic intensity increased slightly but statistically significant from baseline during MAP BP among both black (1.95 vs. 2.07) and white (1.40 vs. 1.55) adults. More non-Hispanic white than non-Hispanic black adults had untreated HTN, whereas more non-Hispanic black than non-Hispanic white adults were prescribed ≥3 BP medications. The decline in systolic BP after adding an antihypertensive medication class and 30-day follow-up for uncontrolled HTN was greater in non-Hispanic white than non-Hispanic black adults.

**Table 3.** Measure Accurately, Act Rapidly, Partner with Patients Metrics in Non-Hispanic Black and Non-Hispanic White Adults at Baseline and After 6 Months of Measure Accurately, Act Rapidly, Partner with Patients Blood Pressure

	Non-Hispanic black adults (*N*=8332)		Non-Hispanic white adults (*N*=3583)	
Metric	Baseline	MAP BP	Baseline	MAP BP
HTN control, %	61.7% [60.68, 62.77]	73.8% [72.81, 74.70]	67.9% [66.35, 69.41]	78.4% [77.02, 79.72]
Repeat BP, %	42.7% [42.45, 43.89]	49.1% [47.70, 50.49]	31.6% [29.72, 33.42]	47.7% [45.50, 49.99]
Therapeutic intensity, *N*	1.95 [1.92, 1.98]	2.07 [2.03, 2.11]	1.40 [1.36, 1.45]	1.55 [1.49, 1.60]
No BP medication, %	5.3% [4.84, 5.80]	4.4% [3.99, 4.87]	8.2% [7.33, 9.13]	7.3% [6.43, 8.14]
1–2 BP medications, %	47.8% [46.76, 47.93]	45.7% [44.66, 46.80]	58.2% [56.60, 59.83]	55.8% [54.19, 57.45]
≥3 BP medications, %	46.9% [45.78, 47.93]	49.8% [48.77, 50.92]	33.6% [32.00, 35.09]	36.9% [35.32, 38.48]
ΔSBP Rx Int, mmHg	13.5 [12.68, 14.36]	16.3 [15.35, 17.35]	15.0 [13.56, 16.39]	18.4 [16.86, 19.87]
30-Day follow-up, %	22.3% [22.09, 23.86]	28.6%27.93, 30.14]	24.8% [23.07, 26.58]	30.3% [28.15, 32.47]

Data are reported as percent or mean and 95% confidence intervals; Therapeutic intensity, number of antihypertensive medication classes ≥standard dose when BP was ^3^140 systolic or ^3^90 diastolic; ΔSBP (mm Hg) after Rx Int, fall in systolic BP after treatment intensification.

Antihypertensive medication classes and most prescribed medications within each class to all adults with HTN (Table 4) as well as a frequency histogram (Fig. 3) of medication classes prescribed to adults with uncontrolled HTN were provided at the system, clinic, and clinician levels to facilitate appropriate treatment intensification. Table 4 included the standard or half-maximal dose, at which ∼80% of the antihypertensive effect occurs, and the percent of adults with HTN prescribed standard dose or higher for each antihypertensive medication listed.

**Table 4.** Most Prescribed Antihypertensive Medication Classes and Specific Medications Prescribed for Adults with Hypertension (*N*=12,370)

Medication class subclass	Generic name	Patients, *n* (%)	Standard dose (mg/day)	Standard dose, *n* (%)
RASB		8498 (68.7)		
ACEI	Lisinopril	3586 (29.0)	20	2209 (61.6)
ARB	Losartan	3240 (26.2)	50	2911 (89.8)
ARB	Valsartan	846 (6.8)	160	629 (74.5)
ACEI	Benazepril	755 (6.1)	20	578 (76.7)
Diuretics		7857 (63.5)		
Thiazide	HCTZ	6152 (49.7)	25	4087 (66.4)
Loop	Furosemide	1767 (14.3)	40	905 (51.5)
Aldo Ant	Spironolactone	643 (5.2)	25	633 (98.8)
CCB		6454 (52.2)		
Dihydropyridine	Amlodipine	5741 (46.4)	5	5431 (94.6)
β-blockers		4148 (33.5)		
β_1_-blocker	Metoprolol	2459 (19.9)	100	881 (35.9)
α,β-blocker	Carvedilol	1030 (8.3)	25	594 (57.8)

RASB, renin–angiotensin system blockers include: ACEI, angiotensin converting enzyme inhibitor; ARB, angiotensin receptor blocker. Diuretics include: HCTZ, hydrochlorothiazide; Aldo ant, aldosterone antagonist. CCB, calcium channel blocker.

## Discussion

HopeHealth, located in the buckle of the stroke belt, serves a diverse population with historically high risk for HTN and cardiovascular events.^1–6^ In fact, 46% of adults seen during the 1-year baseline period before implementing MAP BP had an HTN diagnosis. Before discussions with the AMA, HopeHealth established goals to raise HTN control to ≥75% and improve racial equity in control. To facilitate goal attainment, HopeHealth collaborated with the AMA on MAP BP.^8,9^

During the 6-month MAP BP program, the first goal was attained as BP control to <140/<90 rose from 63.6% at baseline to 75.1% for patients qualifying for the main analysis. Changes in MAP BP process metrics drive improvements in HTN control. Regarding Measure accurately, the mean decline in systolic BP with repeated measurements was similar during baseline and MAP BP. However, the increased frequency of repeat BP measurements contributed to lower BP and improved control.

Act rapidly, or the number of antihypertensive medication classes prescribed at standard dose or higher to adults with uncontrolled BP, rose modestly from 1.8 to 1.9. Roughly two-thirds of adults with uncontrolled HTN were not prescribed three different antihypertensive medication classes at standard dose or higher, the minimum required for apparent treatment-resistant HTN.^10,11^

With regard to Partner metrics, the mean fall in systolic BP following addition of an antihypertensive medication for uncontrolled HTN increased, which suggests greater patient engagement in obtaining and taking the additional medication and possibly prescribing more effective classes or combinations.^22^ The proportion of adults who had a follow-up encounter within 30 days of uncontrolled BP rose modestly and significantly from 23% to 29%.^17^ Thus, both Partner components of MAP BP contributed to the fall in BP and rise in control.

The second goal to improve racial equity in HTN control was unmet. HTN control improved significantly in non-Hispanic black and non-Hispanic white adults (Table 3). The improvement in HTN control was not significantly greater in non-Hispanic black than in non-Hispanic white adults (12.0% vs. 10.4%, *p*=0.16), and a disparity persisted (73.8% vs. 78.4%, *p*<0.0001).

A racial comparison of process metrics may inform efforts to improve equity in HTN control. At baseline, the Measure accurately process was performed more often in non-Hispanic black than in non-Hispanic white adults. The gap closed during MAP BP but remained slightly higher in non-Hispanic black than in non-Hispanic white adults. While opportunity for improvement exists, repeat BP measurement did not explain disparities in control.

Act rapidly, assessed by therapeutic intensity, was greater in black than in white adults at baseline and increased similarly in both groups during MAP BP. Our previous research suggested that black adults had less access to prescribed antihypertensive medication at civilian practice sites, which contributed to higher BP and less control in the former, despite prescription of more antihypertensive medications.^23^ The observation that systolic BP fell less in non-Hispanic black than in non-Hispanic white adults following treatment intensification during the MAP BP program in the current report is consistent with differential access to medications. Follow-up within 30 days of an encounter with uncontrolled HTN was slightly lower in non-Hispanic black than in non-Hispanic white adults, while both groups improved during MAP BP.

The observations suggest that increasing access to antihypertensive medications and follow-up frequency for uncontrolled HTN in black adults could improve equity in HTN control. Since black adults have severe HTN more often than white adults and can encounter more structural barriers to healthy lifestyles and medical care,^24^ greater performance on process metrics in black than white adults may be required to attain equity in control.

There is growing attention to structural racism, rather than biology, as the driver of disparate health outcomes between black and white adults.^24–28^ FQHCs began as a key initiative to mitigate health disparities.^29^ The mean difference in HTN control between non-Hispanic black and non-Hispanic white adults in this study was 4.6%, which is substantially less than the 15% mean difference between non-Hispanic black and non-Hispanic white adults with treated HTN in the civilian population during 2017–2018.^30^ The observations suggest that HopeHealth is reducing health disparities, while striving to eliminate them.

### Health equity implications

MAP BP contributed to the goal of controlling HTN in 75% of eligible adults. MAP BP was more effective when adults with HTN have more visit opportunities to derive program benefits. To reach those with inadequate follow-up, HopeHealth is assessing the value of telemedicine and self-monitored BP using the MAP BP framework.^31,32^

To better address Act rapidly, HopeHealth plans to add clinical pharmacists to team-based care for HTN to facilitate appropriate pharmacotherapy and provide the resources and supports, for example, self-monitored BP and telemedicine, to assist patients in overcoming barriers to BP control. A new hypertension clinic is also planned to begin later in 2022 for patients with uncontrolled and treatment-resistant HTN. To facilitate appropriate pharmacotherapy, the AMA will change the metric from therapeutic intensity to therapeutic intensification or the percentage of encounters with uncontrolled BP at which an antihypertensive medication class is added. The updated metric is applicable to most patients with uncontrolled HTN that is not treatment resistant. The AMA will continue providing training on management of treatment-resistant HTN.^10,11^

## Abbreviations Used

AMA: American Medical Association
AO: automated office
BP: blood pressure
FQHC: federally qualified health center
HTN: hypertension
MAP BP: Measure accurately, Act rapidly, Partner with patients blood pressure
